# Supplementary material for: PRMT5 is an actionable therapeutic target in CDK4/6 inhibitor-resistant ER+/RB-deficient breast cancer
Source: Nat Commun. 2024 Mar 13;15:2287. doi: 10.1038/s41467-024-46495-2 (PMC10937713; doi:10.1038/s41467-024-46495-2)
Supplement: Supplementary file 1 — Supplementary Information [file 41467_2024_46495_MOESM1_ESM.pdf]

Supplementary Table. 1 List of primers

Primers for cloning	Sequence
sgRB1_F1	CACCGCTGAGCGCCGCGTCCAACCG
sgRB1_R1	AAACCGGTTGGACGCGGCGCTCAGC
sgRB1_F2	CACCGCTTCCCGCGTGAGGCGACGG
sgRB1_R2	AAACCCGTCGCCTCACGCGGGAAGC
sgPRMT5_1	tatatatcttgtggaaaggacgaaaCACCGGATGGAAGACAGGCATGCAGGTTTtagagctagaaatagcaagttaaaa
sgPRMT5_2	tatatatcttgtggaaaggacgaaaCACCGACTCCCTCTTGAAACGCGGAGTTTtagagctagaaatagcaagttaaaa
sgPRMT5_3	tatatatcttgtggaaaggacgaaaCACCGATGAACTCCCTCTTGAAACGGTTTtagagctagaaatagcaagttaaaa
shPRMT5_3'UTR_F	CCGGGGCTCAAGCCACCAATCTATGCTCGAGCATAGATTGGTGGCTTGAGCCTTTTT
shPRMT5_3'UTR_R	AATTAAAAAGGCTCAAGCCACCAATCTATGCTCGAGCATAGATTGGTGGCTTGAGCC
PRMT5_E444Q_F	TGCTGACAATcAATTGTCGCC
PRMT5_E444Q_R	AATGAGCCCAGAAGCTCA

Primers for qPCR	Fwd 5'-3'	Rev 5'-3'
ASF1B	CAT GGT AGG TGC AGG TGA TG	AGC AGG GAG ACA CAT GTT TG
CDC25B	CAG AGA CCT GCA TCA GAA GA	ACC CTC ACC CAG ACC AT
CTPS1	GCA CCT GCA TAC AAC CAG AT	CAT CCA CGT CAG TCT AGT TCC
KIF2C	TGA ATC AAC TGC TCT CCA CTG	TCT AGG ACT TGC ATG ATT GCC
KPNA2	ATC AAC AGA CCA ATT TAC AGT GC	AAG GAT GAC CAG ATG CTG AAG
MCM7	CCT TTG ACA TCT CCA TTA GCC T	GGA TGC CAC CTA TAC TTC TGC
MKI67	GAA GCT GGA TAC GGA TGT CA	CGC CTG GTT ACT ATC AAA AGG A
MXD3	GCT TCT CCA GTT CAT TGT GC	ATG GTT ATG CGT CCC TGT G
MYBL2	CTC TCC AGC TCC AAT GTG TC	GAT TCC TGT AAC AGC CTC ACG
TK1	CGT CTC ATC AAC TCT GTG CTT T	GCA TTA ACC TGC CCA CTG T
TOP2A	TGT ACC ATT CAG GCT CAA CAC	GAT TCT GCT AGT CCA CGA TAC ATC
YWHAZ	ACTTTTGGTACATTGTGGCTTCAA	CCGCCAGGACAAAACAGTAT

Supplementary Figure 1.

A

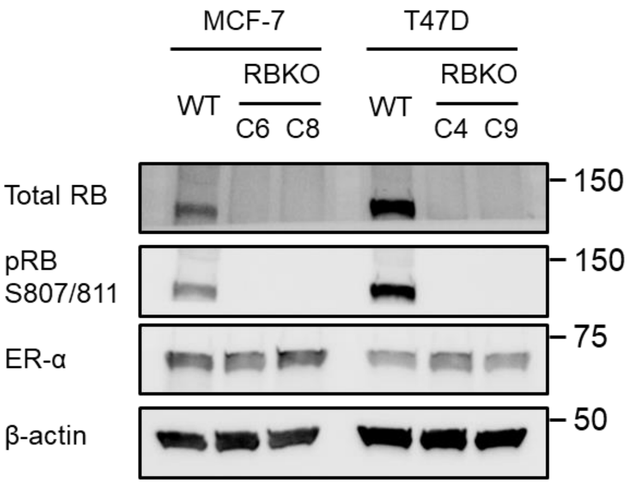

B

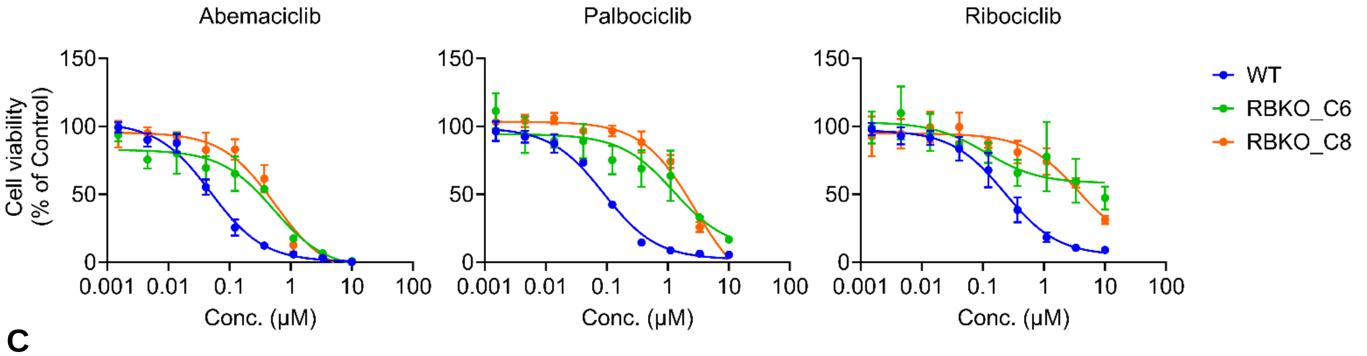

C

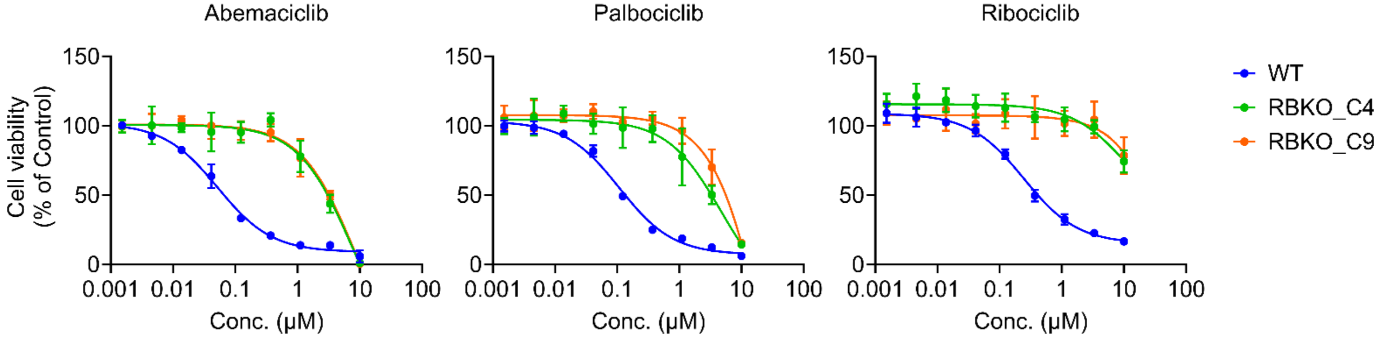

D

IC50<br>μM (fold change)	Abemaciclib	Palbociclib	Ribociclib
WT	0.05 (1)	0.09 (1)	0.24 (1)
RBKO_C6	0.53 (10.6)	1.26 (14.0)	ND
RBKO_C8	0.56 (11.2)	2.80 (31.1)	3.64 (15.8)

E

IC50<br>μM (fold change)	Abemaciclib	Palbociclib	Ribociclib
WT	0.05 (1)	0.11 (1)	0.24 (1)
RBKO_C4	7.59 (151.8)	4.71 (42.8)	ND
RBKO_C9	8.44 (168.8)	32.54 (295.8)	ND

Supplementary Figure 1. *RB1* knockout in ER+ breast cancer cells confers resistance to CDK4/6 inhibitors.

A) Immunoblot analysis of MCF-7 and T47D *RB1* wild-type (WT) and *RB1* knockout (RBKO) cell lysates. The lysates were probed with the indicated antibodies (n = 2 biological replicates). **B-C)** Dose response curves of CDK4/6i. WT (blue) and RBKO (green and orange) cells of MCF-7 **(B)** and T47D **(C)** were treated with a dose range of abemaciclib, palbociclib, or ribociclib for 6 days. Cell viability was measured by the CyQuant assay (mean ± SD, n = 3 biological replicates). **D-E)** IC_{50} of abemaciclib, palbociclib, and ribociclib in WT and RBKO cells of MCF-7 **(D)** and T47D **(E)**. Data represent IC_{50} in µM and fold change in the parenthesis. ND: not determined. Source data are provided as a Source Data file.

Supplementary Figure 2.

A

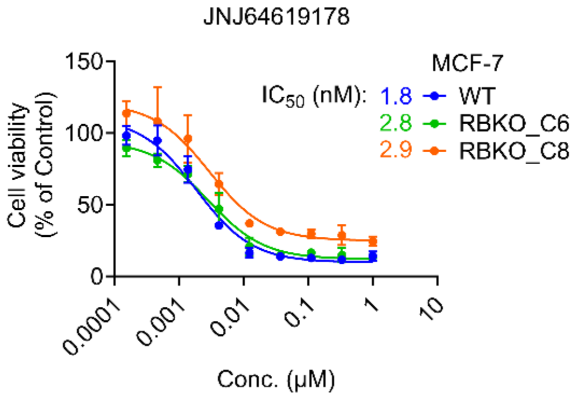

B

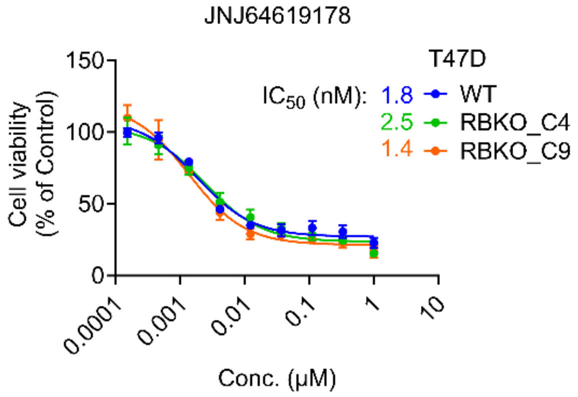

C

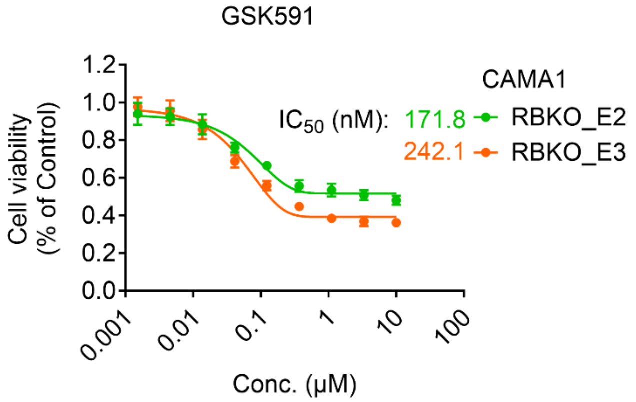

D

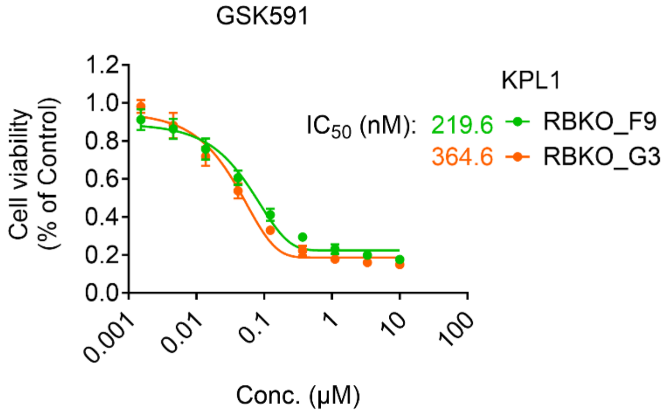

Supplementary Figure 2. Sensitivity of ER+/*RB1* knockout (RBKO) breast cancer cells to PRMT5 inhibitor.

A-B) Dose response curves of JNJ64619178 and **C-D**) GSK591. MCF-7 **(A)** and T47D **(B)** RBKO cells were treated with a concentration range of JNJ64619178 for 6 days. CAMA1 **(C)** and KPL1 **(D)** RBKO cells were treated with a concentration range of GSK591 for 6 days. Cell viability was measured by the CyQuant assay. Data represent mean ± SD (n = 3 biological replicates). Source data are provided as a Source Data file.

Supplementary Figure 3.

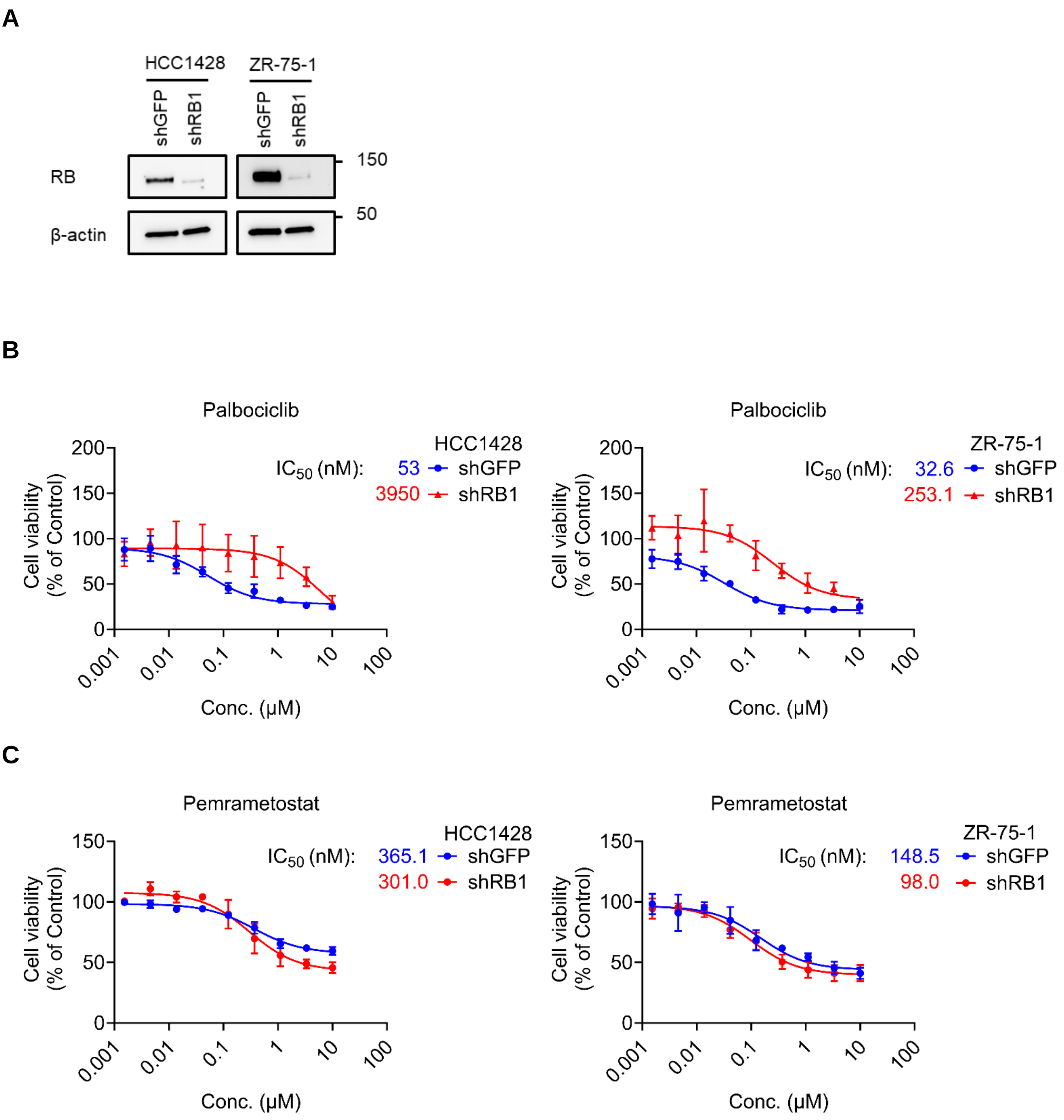

Supplementary Figure 3. Pemrametostat inhibits growth of ER+/RB1 knockdown breast cancer cells.

A) Immunoblot analysis. HCC1428 and ZR-75-1 cells were transcduced with shGFP or shRB1. The lysates were probed with the indicated antibodies (n =2 biological replicates). **B-C)** Dose response curves. Cells were treated with a dose range of palbociclib **(B)** or pemrametostat **(C)** for 6 days. Cell viability was measured by the CyQuant assay (mean ± SD, n = 3 biological replicates). Source data are provided as a Source Data file.

Supplementary Figure 4.

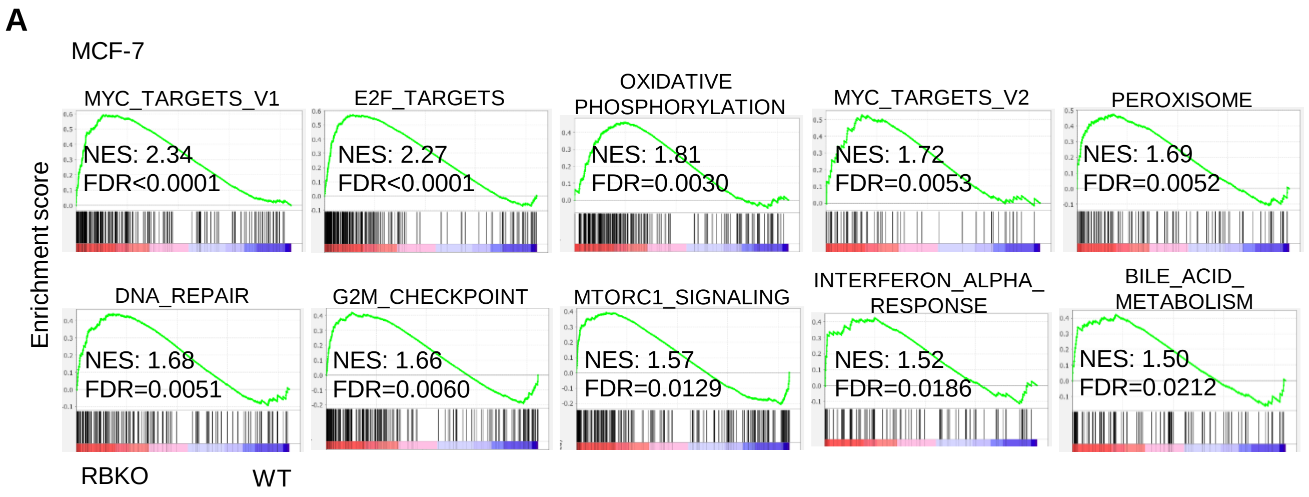

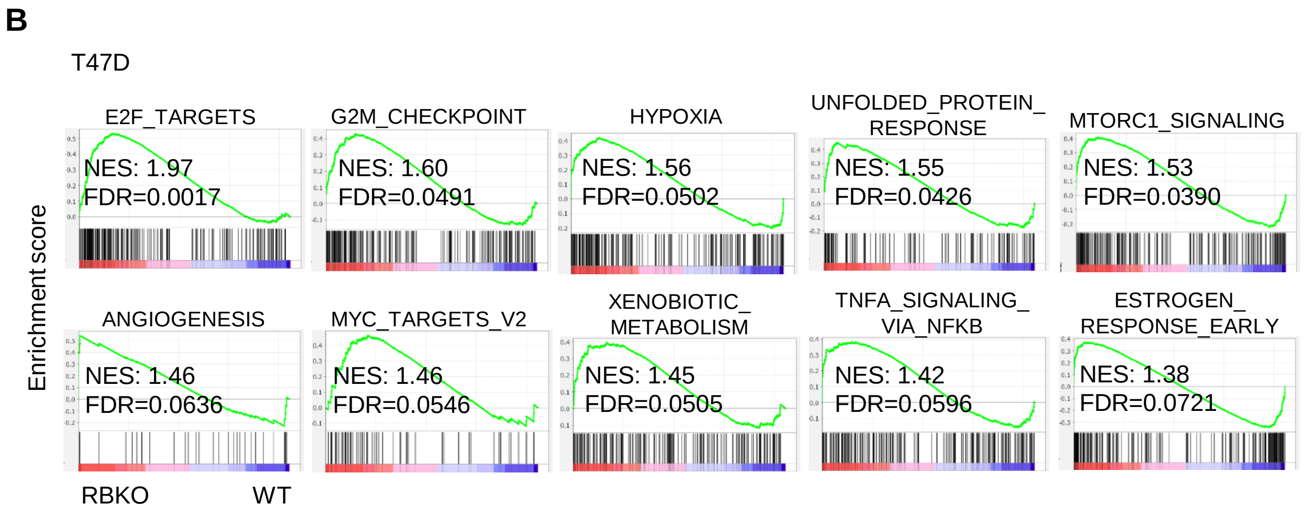

Supplementary Figure 4. Top 10 of upregulated Hallmark gene signatures upon *RB1* knockout.

A-B) Gene set enrichment analysis (GSEA) using RBKO vs WT RNA-seq data from MCF-7 **(A)** and T47D **(B)** cells. NES: normalized enrichment score; FDR: false discovery rate.

Supplementary Figure 5.

A

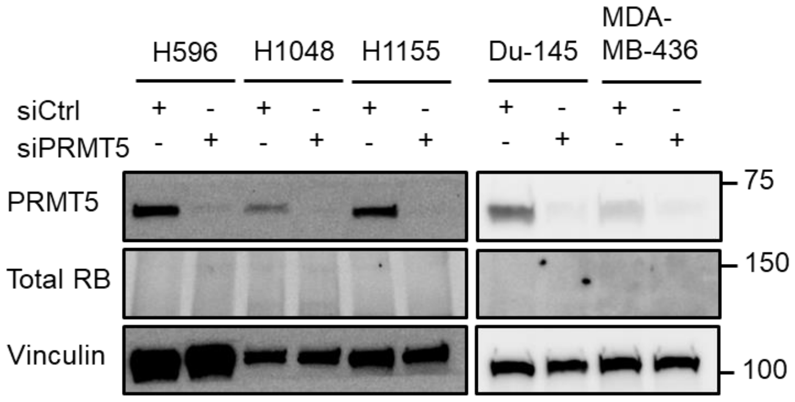

B

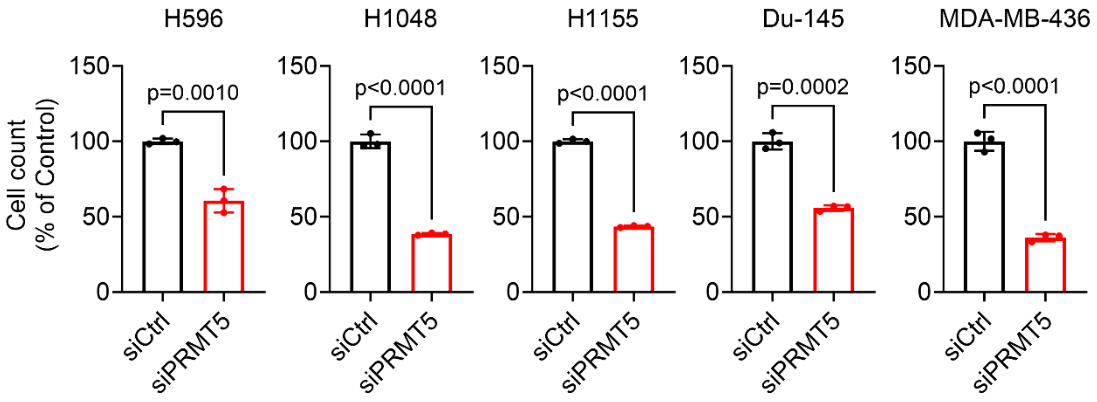

C

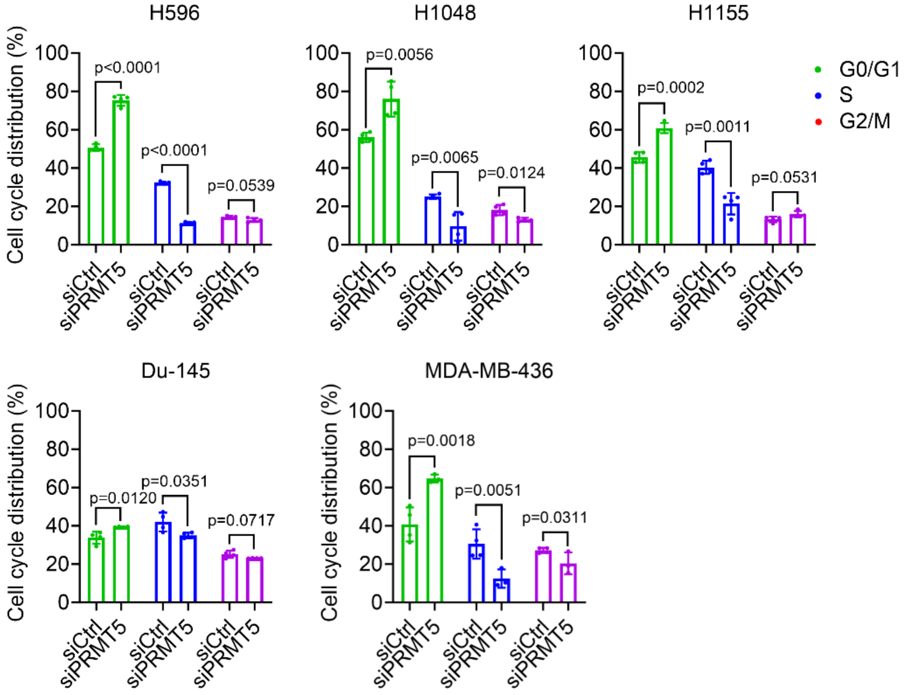

Supplementary Figure 5. *PRMT5* knockdown in *RB1*-deficient cancer cells results in growth inhibition and blocks G1- to S-phase cell cycle progression.

A) Immunoblot analysis of *RB1*-mutant lung cancer (H596, H1048 and H1155), prostate cancer (Du-145) and triple-negative breast cancer (MDA-MB-436) cells. Lysates were collected 3 days after transfection of control siRNA (siCtrl) or a siRNA targeting *PRMT5* (siPRMT5) and then probed with antibodies as indicated (n = 2 biological replicates). **B)** Growth of siCtrl- or siPRMT5-transfected *RB1*-mutant cancer cells. Cell number was counted using a Coulter counterafter 4-6 days after siRNA transfection. Data represent mean ± SD (n = 3 biological replicates), two-sided Student's t test. **C)** Cell cycle analysis. Cells were fixed 3-4 days after transfection of siCtrl or siPRMT5. Cells were stained with propidium iodide (PI) and then analyzed by flow cytometry Data represent mean ± SD (n = 4 biological replicates, MDA-MB-436 siPRMT5 n = 3 biological replicates), two-sided Student's t test. Source data are provided as a Source Data file.

Supplementary Figure 6.

A

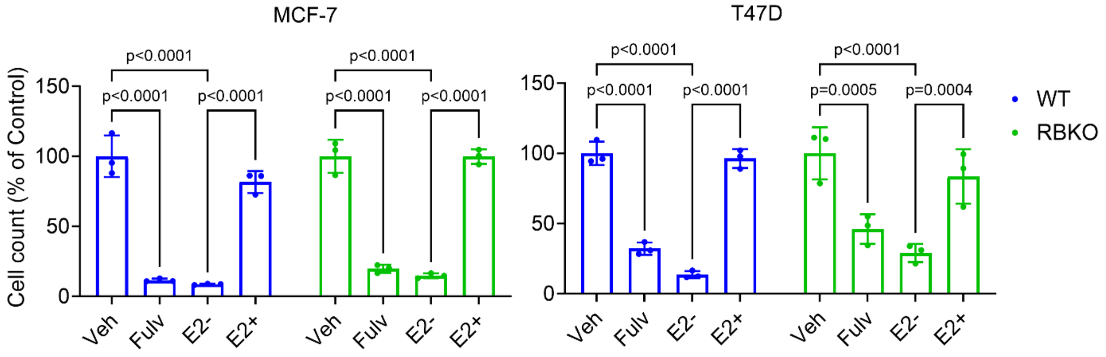

B

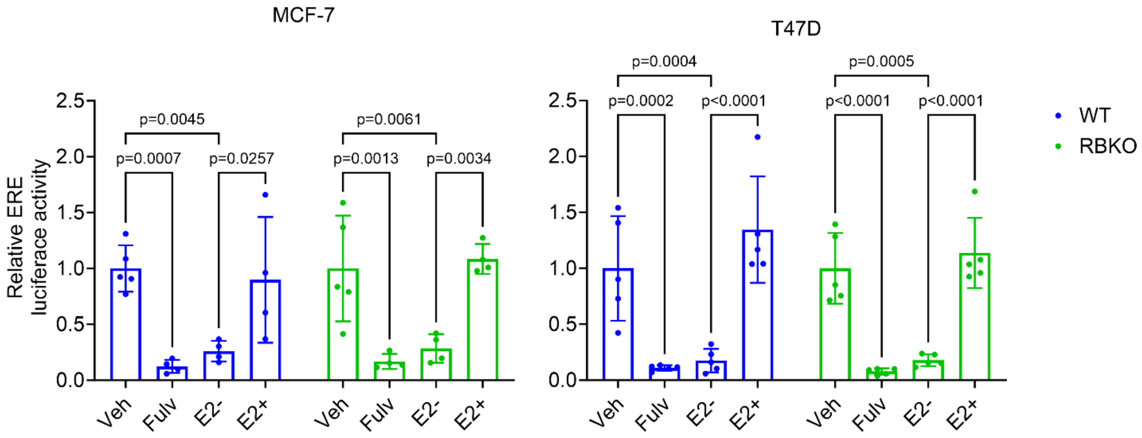

C

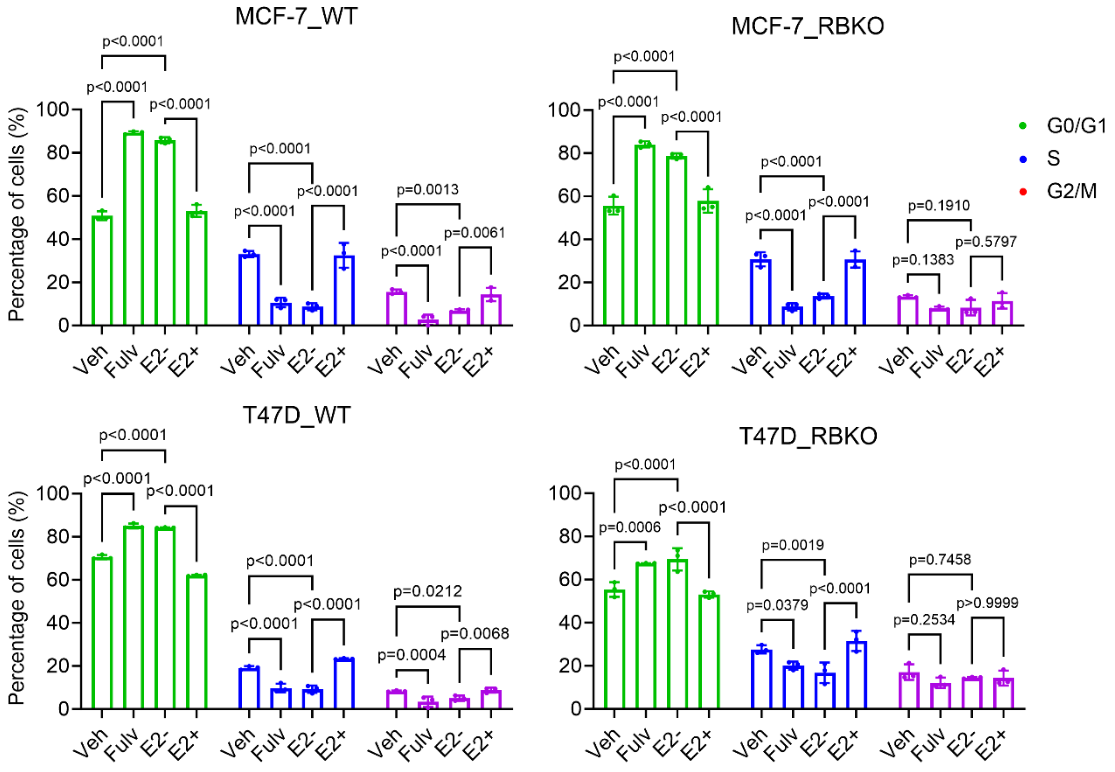

Supplementary Figure 6. ER+ breast cancer cells are sensitive to anti-estrogens irrespective of *RB1* status.

A) Monolayer growth of MCF-7 and T47D WT and RBKO cells treated with vehicle control (Veh), 10 nM fulvestrant (Fulv), E2-deprived IMEM (E2-) and 1 nM 17β-estradiol in E2-deprived IMEM (E2+). Cells were counted on day-7 using a Coulter counter. Data represent mean ± SD (n = 3 biological replicates), one-way ANOVA with a Dunnett's post-hoc test. **B)** Estrogen responsive element (ERE) luciferase reporter assay. Cells were co-transfected with pGLB-MERE and internal control pCMV-Renilla. Next day, MCF-7 and T47D WT and RBKO cells were treated as indicated for 24 hours and then were subjected to dual-luciferase reporter assay. Data represent mean ± SD (n = 5 biological replicates, MCF-7_WT and _RBKO Fulv, E2-, and E2+ n = 4 biological replicates), one-way ANOVA with a Dunnett's post-hoc test. **C)** Cell cycle analysis of MCF-7 and T47D WT and RBKO cells. Cells were treated as indicated for 3 days, fixed, and then stained with propidium (PI). Cell cycle analysis was conducted using flow cytometry (mean ± SD, n = 3 biological replicates), one-way ANOVA with a Dunnett's post-hoc test. Source data are provided as a Source Data file.

Supplementary Figure 7.

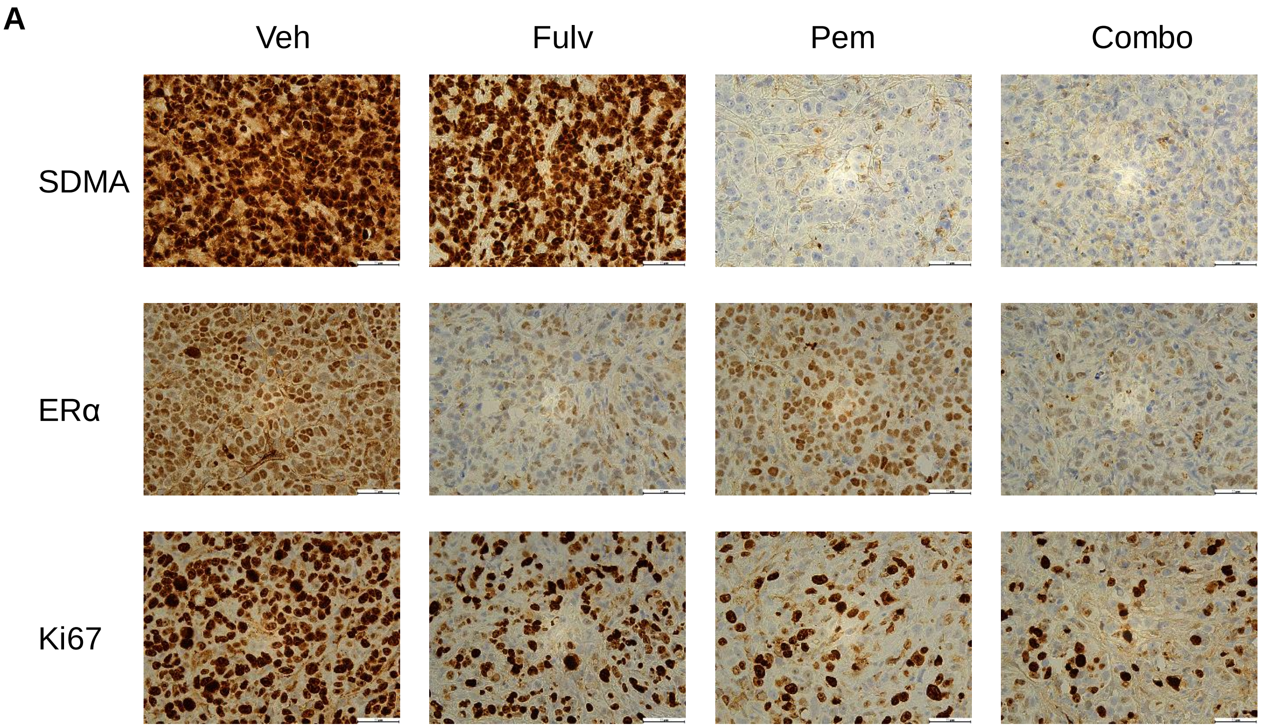

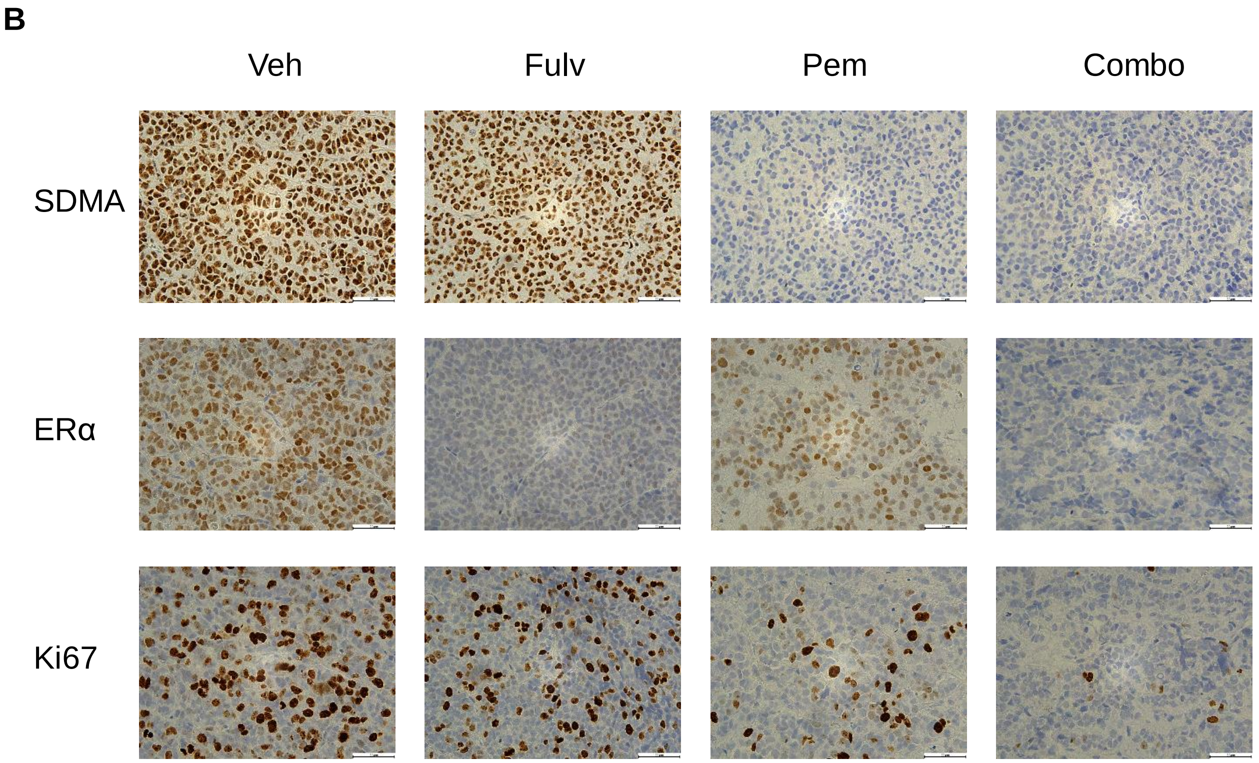

Supplementary Figure 7. IHC of ER+/RB-deficient tumors.

A-B) Representative immunohistochemistry (IHC) images of SDMA, ERα, and Ki67 of MCF-7_RBKO xenografts **(A)** and ER+/*RB1*-deleted PDXs **(B)**. The mice were treated with vehicle control (Veh), fulvestrant (Fulv, 5 mg/kg/week, s.c.), pemrametostat (Pem, 200 mg/kg/day, p.o.), or combination of both drugs (Combo). Scale bars on the lower right depict 50 µM.

Supplementary Figure 8.

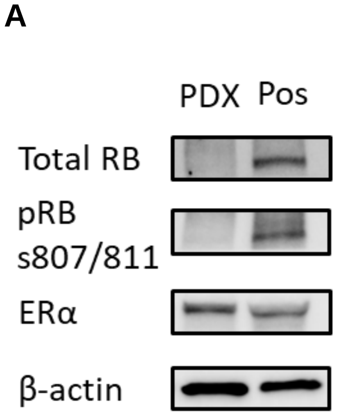

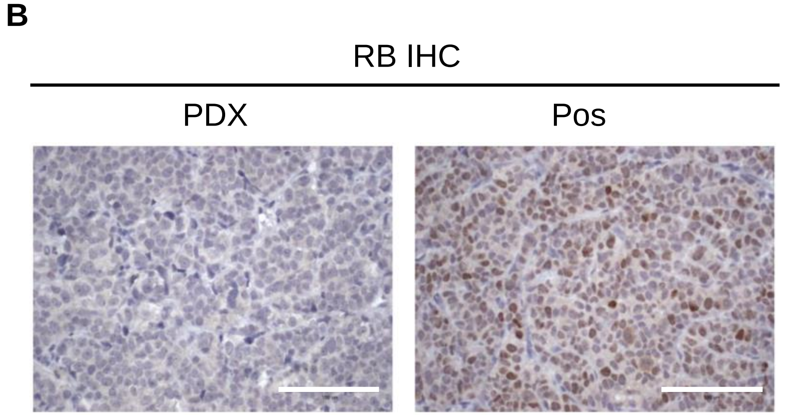

Supplementary Figure 8. Characterization of the ER+/*RB1*-deleted PDX.

A) Immunoblot analysis of the patient-derived xenografts (PDX) lysates, probed with the indicated antibodies (n = 2 biological replicates). **B)** IHC of total RB on PDX FFPE sections (n = 2 biological replicates). Pos: positive control. Scale bars on the lower right depict 100 µM.

Supplementary Figure 9.

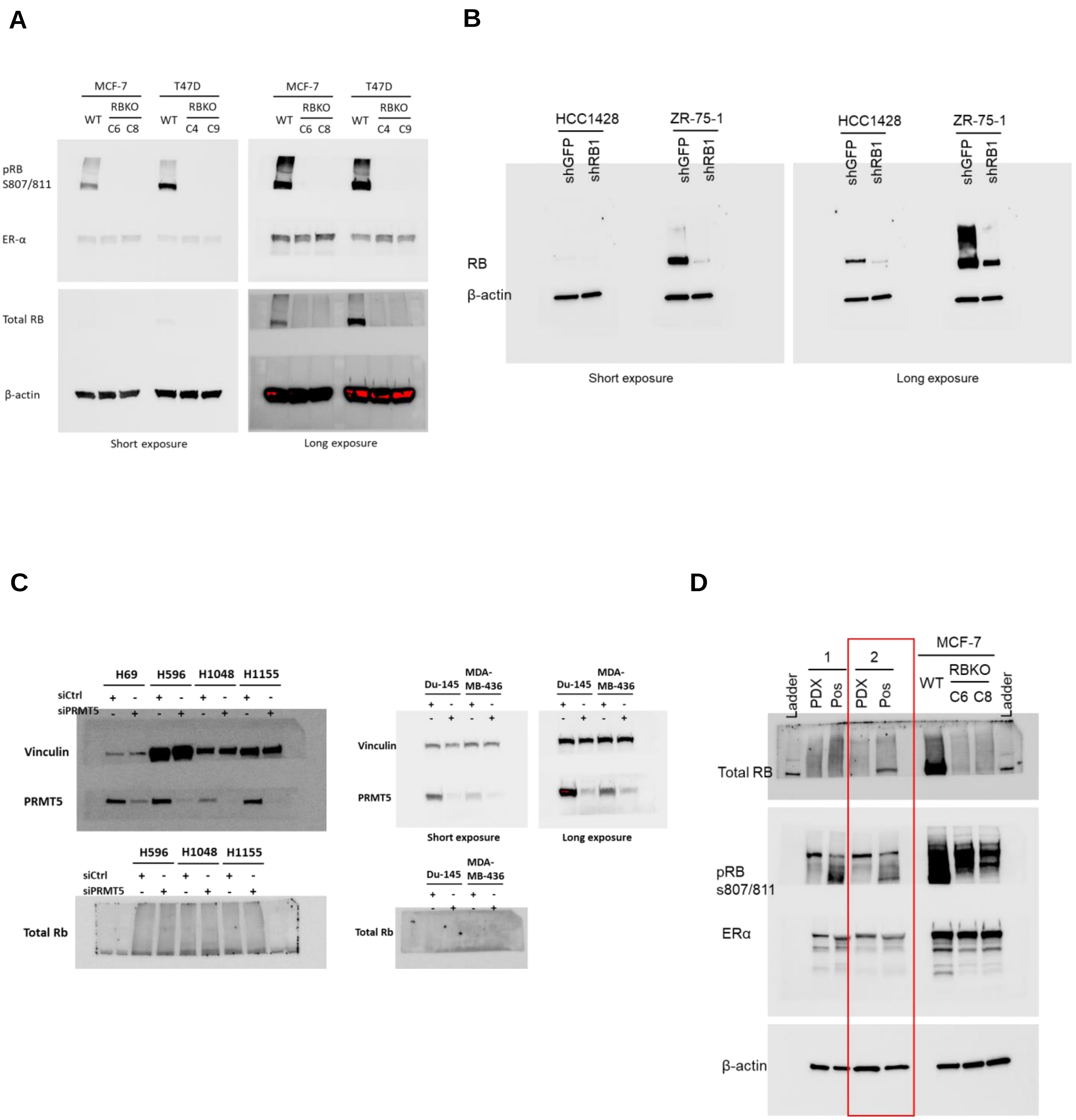

SDS sample buffer can affect immunoblot analysis of Rb. #1 and #2 are the same set of samples extracted using Laemmli (1) and NuPage (2) sample buffer, respectively. NuPage sample buffer (highlighted in red) was selected for all immunoblot analysis in this study after this comparison. Pos: positive control.

Supplementary Figure 9. Uncropped immunoblot images

Uncropped immunoblot images for **A) Supplementary Figure 1A**, **B) Supplementary Figure 3A**, **C)**, **Supplementary Figure 5A**, and **D) Supplementary Figure 7A**.

Supplementary Figure 10.

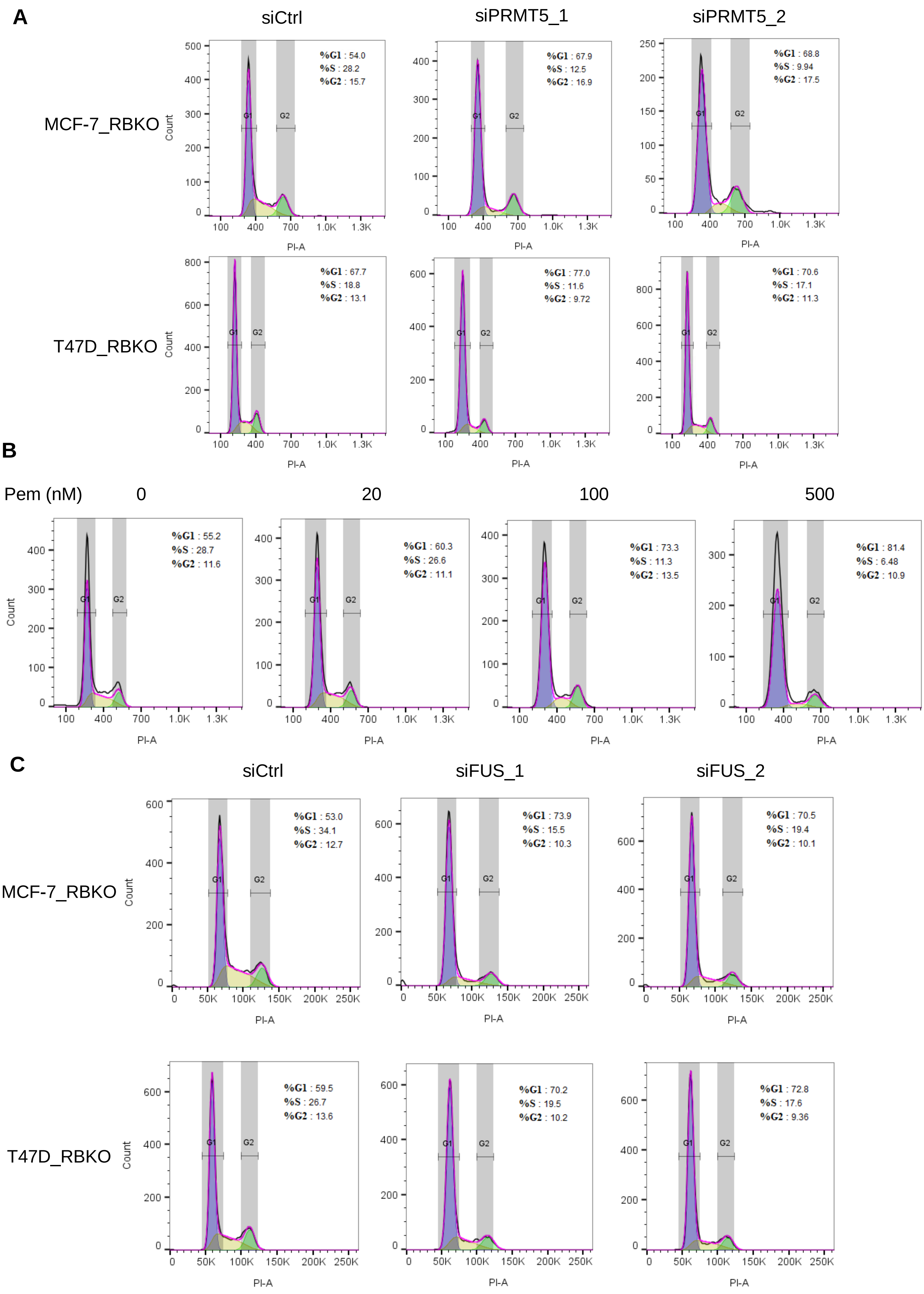

Supplementary Figure 10. Continued

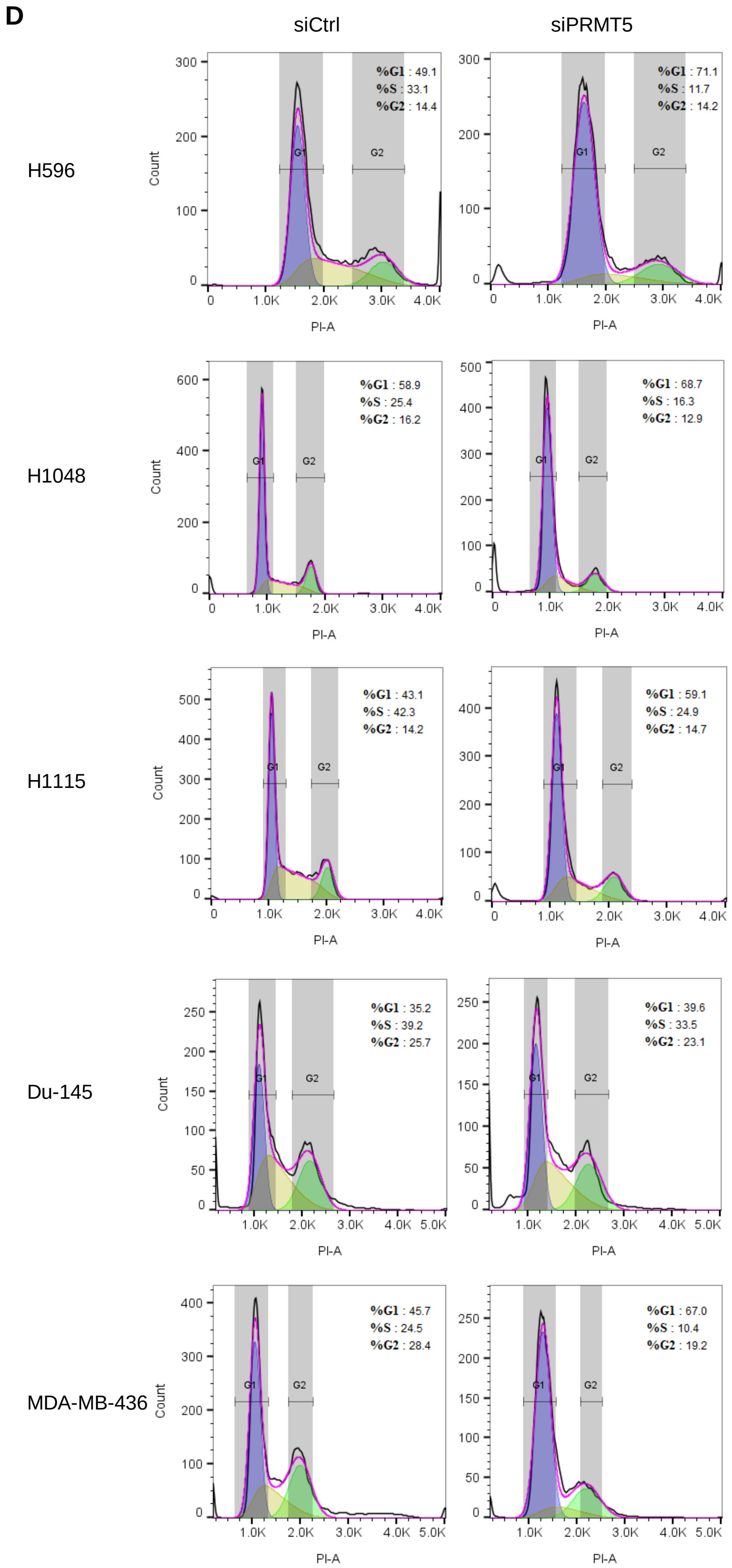

Supplementary Figure 10. Continued

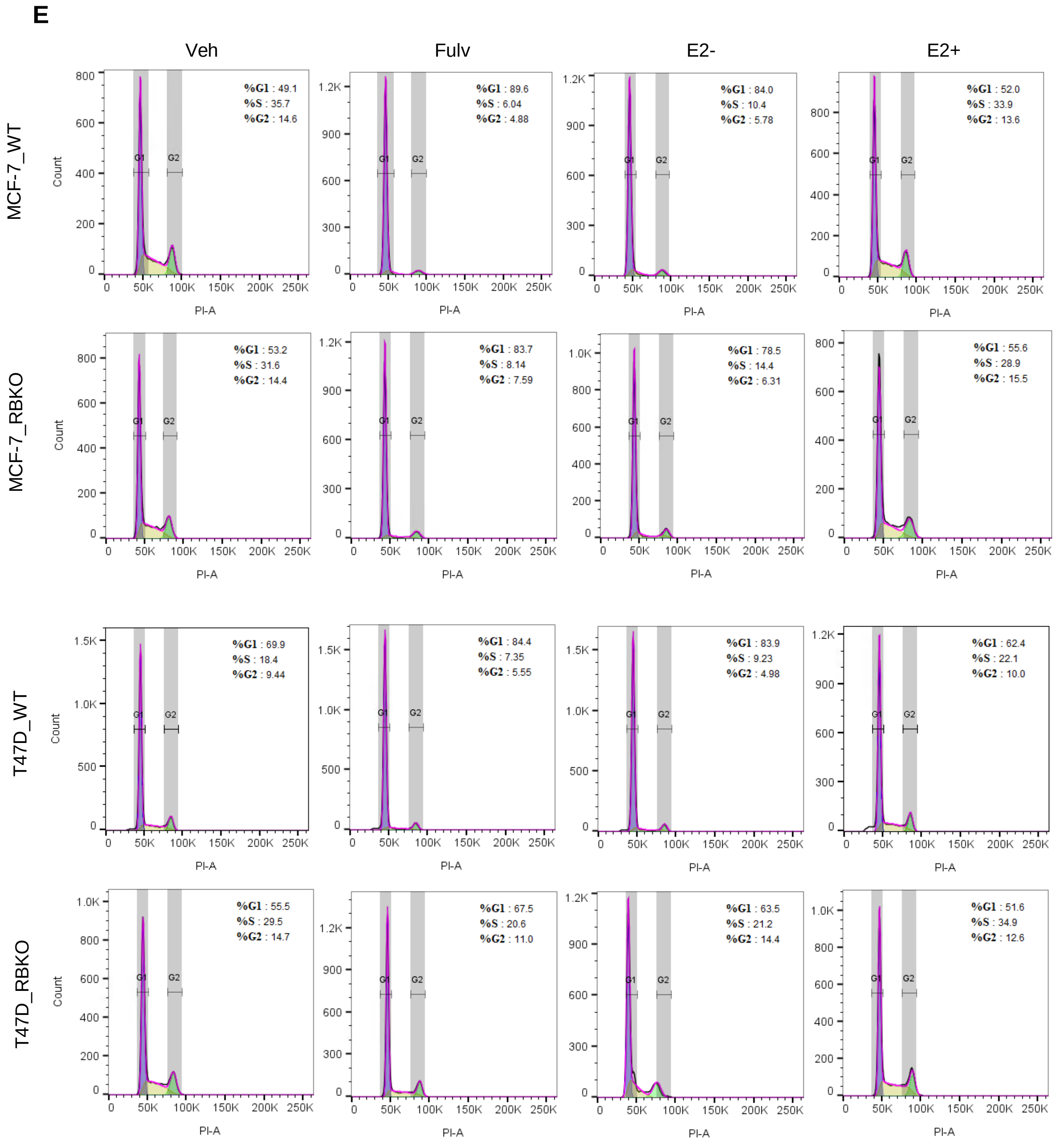

Supplementary Figure 10. Gating strategy for cell cycle analysis
Gating strategy for **A) Fig. 3D**, **B) Fig. 3E**, **C) Fig. 4G**, **D) Supplementary Figure 5C**, and **E) Supplementary Figure 6C.**
