# Supplementary material for: PRMT5 is an actionable therapeutic target in CDK4/6 inhibitor-resistant ER+/RB-deficient breast cancer
Source: Nat Commun. 2024 Mar 13;15:2287. doi: 10.1038/s41467-024-46495-2 (PMC10937713; doi:10.1038/s41467-024-46495-2)
Supplement: Supplementary file 2 — Reporting Summary [file 41467_2024_46495_MOESM2_ESM.pdf]

nature portfolio

Corresponding author(s): Carlos L. Arteaga

Last updated by author(s): Jan 27, 2024

Reporting Summary

Nature Portfolio wishes to improve the reproducibility of the work that we publish. This form provides structure for consistency and transparency in reporting. For further information on Nature Portfolio policies, see our Editorial Policies and the Editorial Policy Checklist.

Statistics

For all statistical analyses, confirm that the following items are present in the figure legend, table legend, main text, or Methods section.

n/a	Confirmed	
☐	☒	The exact sample size (*n*) for each experimental group/condition, given as a discrete number and unit of measurement
☐	☒	A statement on whether measurements were taken from distinct samples or whether the same sample was measured repeatedly
☐	☒	The statistical test(s) used AND whether they are one- or two-sided<br>*Only common tests should be described solely by name; describe more complex techniques in the Methods section.*
☒	☐	A description of all covariates tested
☐	☒	A description of any assumptions or corrections, such as tests of normality and adjustment for multiple comparisons
☐	☒	A full description of the statistical parameters including central tendency (e.g. means) or other basic estimates (e.g. regression coefficient) AND variation (e.g. standard deviation) or associated estimates of uncertainty (e.g. confidence intervals)
☐	☒	For null hypothesis testing, the test statistic (e.g. *F*, *t*, *r*) with confidence intervals, effect sizes, degrees of freedom and *P* value noted<br>*Give P values as exact values whenever suitable.*
☒	☐	For Bayesian analysis, information on the choice of priors and Markov chain Monte Carlo settings
☒	☐	For hierarchical and complex designs, identification of the appropriate level for tests and full reporting of outcomes
☒	☐	Estimates of effect sizes (e.g. Cohen's *d*, Pearson's *r*), indicating how they were calculated

Our web collection on statistics for biologists contains articles on many of the points above.

Software and code

Policy information about availability of computer code

Data collection	Microsoft Excel 2016
Data analysis	One-way ANOVA and two-sided Student's t test were conducted using GraphPad Prism ver. 9. Gene set enrichment analysis (GSEA) and gene ontology (GO) analysis were conducted using the GSEA software ver. 4.0.3 downloaded from Broad Institute (https://www.gsea-msigdb.org/gsea/downloads.jsp) and DAVID (https://david.ncifcrf.gov/home.jsp), respectively. Differential gene expression analysis was conducted using DESeq2. RNA splicing analysis was conducted using SUPPA2 ver. 2.2 and IRFinder ver. 1.3. Mass spectrometry data analysis was conducted using Proteome Discoverer 2.4 and were searched using the human protein database from UniProt. Quantification of immunoblot analysis was conducted using Image J ver. 1.53. Flowjo ver. 10 was used to analyze flow cytometry results. ChIP-seq was analyzed using fastqc ver. 0.11.8 and MACS2 ver. 2.1.0. Deeptools ver. 2.4.2 was used to generate BigWig files.

For manuscripts utilizing custom algorithms or software that are central to the research but not yet described in published literature, software must be made available to editors and reviewers. We strongly encourage code deposition in a community repository (e.g. GitHub). See the Nature Portfolio guidelines for submitting code & software for further information.

Data

Policy information about availability of data

All manuscripts must include a data availability statement. This statement should provide the following information, where applicable:

- Accession codes, unique identifiers, or web links for publicly available datasets
- A description of any restrictions on data availability
- For clinical datasets or third party data, please ensure that the statement adheres to our policy

All data associated with this study are presented in the paper or Supplementary Information. Source data are provided as a Source Data file. Raw RNA-seq and ChIP-seq data generated in this study have been deposited in the Gene Expression Omnibus under accession code GSE236500 (https://www.ncbi.nlm.nih.gov/geo/query/acc.cgi?acc=GSE236500). Raw MS data generated in this study were deposited in the ProteomeXchange Consortium via the PRIDE partner repository under accession code PXD046996 (https://proteomecentral.proteomexchange.org/ui?search=PXD046996). Human reference genome assembly GRCh37 and GRCh38 were used for data analysis.

Research involving human participants, their data, or biological material

Policy information about studies with human participants or human data. See also policy information about sex, gender (identity/presentation), and sexual orientation and race, ethnicity and racism.

Reporting on sex and gender	De-identified tissue biopsy was collected from one female patient. Sex and gender were not considered for the study design and sample collection. A written informed consent for the purpose of generating patient-derived xenografts was obtained. No sex- and gender-based analysis were conducted in this study.
Reporting on race, ethnicity, or other socially relevant groupings	No patient data analysis was conducted in this study.
Population characteristics	No patient data analysis was conducted in this study.
Recruitment	The patient was recruited according to the study protocol BRE03103 (https://clinicaltrials.gov/study/NCT00899301). No self-selection bias or other biases were presented to impact results.
Ethics oversight	The study was approved under the study protocol BRE03103 (https://clinicaltrials.gov/study/NCT00899301) with IRB approval number 030747 at Vanderbilt University.

Note that full information on the approval of the study protocol must also be provided in the manuscript.

Field-specific reporting

Please select the one below that is the best fit for your research. If you are not sure, read the appropriate sections before making your selection.

[x] Life sciences   [ ] Behavioural & social sciences   [ ] Ecological, evolutionary & environmental sciences

For a reference copy of the document with all sections, see nature.com/documents/nr-reporting-summary-flat.pdf

Life sciences study design

All studies must disclose on these points even when the disclosure is negative.

Sample size	In general, sample size was determined based on standards for cell line and animal studies from our previous published studies (PMID: 33127913). We have attempted to have a minimum of three biological replicates with sufficient reproducibility if not otherwise specified. The number of replicates is shown in the figure legends.
Data exclusions	No data were excluded from the analysis.
Replication	All experiments were conducted at least two independent times with similar results if not otherwise specified. Therefore, all attempts at replication were successful.
Randomization	For all experiments, subjects were randomly assigned to experimental groups.
Blinding	For IHC quantification analysis, a breast cancer pathologist was blinded to treatment groups and performed quantification for the staining. Blinding was not possible for other experiments as the investigators must be aware of what treatment to give cells and animals.

Reporting for specific materials, systems and methods

We require information from authors about some types of materials, experimental systems and methods used in many studies. Here, indicate whether each material, system or method listed is relevant to your study. If you are not sure if a list item applies to your research, read the appropriate section before selecting a response.

Materials & experimental systems

n/a	Involved in the study
	☒ Antibodies
	☒ Eukaryotic cell lines
☒	Palaeontology and archaeology
	☒ Animals and other organisms
☒	Clinical data
☒	Dual use research of concern
☒	Plants

Methods

n/a	Involved in the study
	☒ ChIP-seq
	☒ Flow cytometry
☒	MRI-based neuroimaging

Antibodies

Antibodies used

For immunoblot analysis: Antibodies purchased from Cell Signaling include: PRMT5 Ab (2252s, 1:1000), Total Rb (4H1) mAb (9309s, 1:1000), pRB/s807/811 (D20B12) mAb (8516s, 1:1000), β-actin (13E5) mAb (4970s, 1:5000), Vinculin (E1E9V) mAb (13901s, 1:2000), SDMA Ab (13222s, 1:1000), MEP50 Ab (2823s, 1:1000), PRMT5 (D5P2T) mAb (79998s, 1:1000), and ORC2 (3G6) mAb (4736S, 1:1000); Antibodies purchased from Santa Cruz Biotechnology include: ERα (F-10) mAb (sc-8002, 1:1000) and POLE (D-10) mAb (sc-390785, 1:1000); Antibodies purchased from Abcam: PRMT5 (EPR5772) mAb (ab109451, 1:1000) and GINS1 (EPR13359) mAb (ab181112, 1:1000); FUS Ab was purchased from Proteintech (11570-1-AP, 1:1000); Total Pol II (4H8) mAb was purchased from MilliporeSigma (05-623, 1:1000); APC7 Ab was purchased from Bethyl Laboratories (A302-551A, 1:1000). For Co-IP: PRMT5 mAB (EPR5772) was purchased from Abcam (ab109451, 1:250); FUS mAB (4H11) was purchased from Santa Cruz Biotechnology (sc-47711, 1:100). For ChIP-seq: pSer2 Pol II Ab was purchased from (ab5095, 1:40). For IHC, SDMA Ab was purchased from Cell Signaling (13222s, 1:600); ERα mAb (F-10) was purchased from Santa Cruz Biotechnology (sc-8002, 1:800); Ki67 (MIB-1) mAb was purchased from Agilent (IR62661-2, ready to use).

Validation

PRMT5 Rabbit antibody (Cell Signaling, 2252s) detects endogenous levels of total PRMT5 protein. The antibody does not cross-react with other related PRMT proteins. This antibody has been validated for immunoblot analysis in previous reports (e.g., PMID: 34518531 and 34330913).
Rb (4H1) Mouse mAB (Cell Signaling, 9309s) detects endogenous levels of total Rb protein. The antibody does not cross-react with the Rb homologues p107 or p130, or with other proteins. This antibody has been validated for immunoblot analysis in previous reports (e.g., PMID: 36719686 and 36602226).
Phospho-Rb (Ser807/811) (D20B12) XP® Rabbit mAb (Cell Signaling, 8516s) recognizes endogenous levels of Rb protein only when phosphorylated at Ser807, Ser811, or at both sites. This antibody does not cross-react with Rb phosphorylated at Ser608. This antibody has been validated for immunoblot analysis in previous reports (e.g., PMID: 36047562 and 36207346).
β-Actin (13E5) Rabbit mAb (Cell Signaling, 2252s) detects endogenous levels of total β-actin protein. Depsite the high sequence identity between the cytoplasmic actin isoforms, β-actin and cytoplasmic γ-actin, β-Actin (13E5) rabbit mAb does not cross-react with cytoplasmic γ-actin, or any other actin isoforms.
Vinculin (E1E9V) XP® Rabbit mAB antibody (Cell Signaling, 13901s) recognizes endogenous levels of total vinculin protein. This antibody also reacts with metavinculin, a 145 kDa splice variant of vinculin. This antibody has been validated for immunoblot analysis in previous reports (e.g., PMID: 36512423 and 36647821).
Symmetric Di-Methyl Arginine Motif [sdme-RG] MultiMab™ Rabbit mAb (Cell Signaling, 13222s) mix recognizes endogenous levels of proteins that are symmetrically dimethylated on arginine residues. This antibody does not cross-react with monomethylated, asymmetrically methylated arginine, or methylated lysine residues. This antibody has been validated for immunoblot analysis and IHC (e.g., PMID: 36841868).
MEP50 Rabbit antibody (Cell Signaling, 2823s) detects endogenous levels of total MEP50 protein. This antibody has been validated for immunoblot analysis (e.g., PMID: 36167829)
ORC2 (3G6) Rat mAb (Cell Signaling, 4736s) recognizes endogenous levels of total ORC2 protein. The antibody does not cross-react with other ORC subunits. This antibody has been validated for immunoblot analysis (e.g., PMID: 24989122).
ERα (F-10) Mouse mAB (Santa Cruz Biotechnology, sc-8002) is specific for an epitope mapping between amino acids 576-595 at the C-terminus of Estrogen Receptor alpha of human origin. This antibody has been validated for immunoblot analysis (e.g., PMID: 36749874).
POLE (D-10) Mouse mAB (Santa Cruz Biotechnology, sc-390785) was raised against amino acids 1-300 mapping at the N-terminus of DNA pol ε A of human origin. This antibody has been validated for immunoblot analysis (e.g., PMID: 36402816).
GINS1 (EPR13359) is a Rabbit mAb (Abcam, ab181112). This antibody has been validated for immunoblot analysis (e.g., PMID: 32769987).
FUS antibody (Proteintech, 11570-1-AP) is a rabbit polyclonal antibody raised against an internal region of human FUS. This antibody has been validated for immunoblot analysis (e.g., PMID: 35075293).
Total Pol II (4H8) Mouse mAb (MilliporeSigma, 05-623) recognizes peptide containing 10 repeats of YSPT[pS]PS corresponding to the carboxyl-terminal domain of RNA polymerase II. This antibody has been validated for immunoblot analysis (e.g., PMID:24910128).
APC7 Rabbit Ab (Bethyl Laboratories, A302-551A) recognizes a region between residue 75 and 125 of human anaphase promoting complex subunit 7 using the numbering given in entry AAI41849. 1 (GeneID 51434).
PRMT5 (EPR5772) Rabbit mAB (Abcam, ab109451). This antibody has been validated for Co-IP(e.g., PMID: 35655230).
FUS (4H11) Mouse mAB (Santa Cruz Biotechnology, sc-47711) was raised against a fusion protein corresponding to the C-terminus of human FUS. This antibody has been validated for Co-IP (e.g., PMID:PMID: 36261283).
ChIP grade pSer2 Pol II Rabbit Ab (Abcam, ab5095) recognizes the phosphorylated serine found in the amino acid 2 position of the C-terminal domain repeat YSPTSPS. This antibody has been validated for ChIP-seq (e.g., PMID: 35115715 and 35614107).
Ki67 (MIB-1) Mouse mAb (Agilent, IR62661-2) recognizes recombinant peptide corresponding to a 1002 bp Ki-67 cDNA fragment. This antibody is ready-to-use and provided in liquid form in a buffer containing stabilizing protein and 0.015 mol/L sodium azide. This antibody has been validated for IHC (e.g., PMID: 7685843).

Eukaryotic cell lines

Policy information about cell lines and Sex and Gender in Research

Cell line source(s)	MCF-7 (Cat. No. HTB-22), T47D (Cat. No. HTB-133), HCC1428 (Cat. No. CRL-2327), ZR-75-1 (Cat. No. CRL-1500), and MDA-MB-436 (Cat. No. HTB-130) cells were purchased from ATCC. 293FT cells were purchased from Invitrogen (Cat. No. R70007). H596, H1048 and H1155 cell lines were kindly provided by Dr. John Minna. Du-145 cells were kindly provided by Dr. Ganesh Raj. CAMA1 and KPL1 cells were kindly provided by Dr. Benjamin Neel.
Authentication	All cell lines were authenticated by the short-tandem repeat (STR) method. The tests were performed on 11/20/23.
Mycoplasma contamination	All cell lines tested negative for mycoplasma contamination.
Commonly misidentified lines (See ICLAC register)	No commonly misidentified cell lines were used in the study.

Animals and other research organisms

Policy information about studies involving animals; ARRIVE guidelines recommended for reporting animal research, and Sex and Gender in Research

Laboratory animals	Six weeks old female athymic nude-Foxn1nu mice and NOD-scid IL2Rgammanull (NSG) mice were purchased from Envigo. All mice housed in barrier facilities were maintained in individually ventilated microisolator cages. All caging equipment was autoclaved and all feed was a commercial irradiated diet. Cage manipulations and animal handling was performed in cage change stations or biosafety cabinets. Automated watering systems provided water that was purified through reverse osmosis and chlorination. The standard white light cycle was from 6:00 AM to 5:59PM and the dark cycle was from 6:00PM to 5:59AM.
Wild animals	No wild animals were used in this study.
Reporting on sex	Breast cancer happens mainly in female (>99%). To better mimic physiological condition and tumor microenvironment of this disease, we used only female mice.
Field-collected samples	No field-collected animals were used in this study.
Ethics oversight	Animal experiments were approved by the UTSW Institutional Animal Care and Use Committee (IACUC, protocol 2018-102359) and Department of the Army, Animal Care and Use Review Office (ACURO, protocol BC210406.e001).

Note that full information on the approval of the study protocol must also be provided in the manuscript.

Plants

Seed stocks	*Report on the source of all seed stocks or other plant material used. If applicable, state the seed stock centre and catalogue number. If plant specimens were collected from the field, describe the collection location, date and sampling procedures.*
Novel plant genotypes	*Describe the methods by which all novel plant genotypes were produced. This includes those generated by transgenic approaches, gene editing, chemical/radiation-based mutagenesis and hybridization. For transgenic lines, describe the transformation method, the number of independent lines analyzed and the generation upon which experiments were performed. For gene-edited lines, describe the editor used, the endogenous sequence targeted for editing, the targeting guide RNA sequence (if applicable) and how the editor was applied.*
Authentication	*Describe any authentication procedures for each seed stock used or novel genotype generated. Describe any experiments used to assess the effect of a mutation and, where applicable, how potential secondary effects (e.g. second site T-DNA insertions, mosiacism, off-target gene editing) were examined.*

ChIP-seq

Data deposition

- [x] Confirm that both raw and final processed data have been deposited in a public database such as GEO.
- [x] Confirm that you have deposited or provided access to graph files (e.g. BED files) for the called peaks.

Data access links *May remain private before publication.*	https://www.ncbi.nlm.nih.gov/geo/query/acc.cgi?acc=GSE236500
Files in database submission	GSM7548835 pSer2 Pol II_DMSO_1<br>GSM7548836 pSer2 Pol II_DMSO_2<br>GSM7548837 pSer2 Pol II_Pem_1<br>GSM7548838 pSer2 Pol II_Pem_2<br>GSM7548839 Input_DMSO<br>GSM7548840 Input_Pem

	Supplementary files:<br>GSE236496_Peak_calls.csv.gz (Broad peak calling)<br>GSE236496_RAW.tar (BigWig files)
Genome browser session<br>(e.g. UCSC)	*Provide a link to an anonymized genome browser session for "Initial submission" and "Revised version" documents only, to enable peer review. Write "no longer applicable" for "Final submission" documents.*

Methodology

Replicates	ChIP-seq was performed in duplicate for each treatment group (DMSO- or pemrametostat-treated MCF-7 RBKO cells).
Sequencing depth	76M to 81.2M/sample. Pair-ended.
Antibodies	ChIP grade pSer2 Pol II antibody (Abcam, ab5095).
Peak calling parameters	macs2 callpeak -t ChIP_Sample_dedup_reads.bam -c Input_dedup_reads.bam --name ChIP_Sample --format BAMPE --broad
Data quality	The quality of the raw data was checked using fastqc (v0.11.8). Peak calling was performed using MACS2 (v 2.1.0) with default parameters (5% FDR) and input DNA as negative control.
Software	The quality of the raw data was checked using fastqc (v0.11.8). Raw reads were mapped to hg19 using BWA (v 0.7.17-r1188). Low quality and duplicated reads were removed using Picard. Peak calling was performed using MACS2 (v 2.1.0) with default parameters and input DNA as negative control. Supplementary_files_format_and_content: Deeptools (2.4.2) was used to generate BigWig files.

Flow Cytometry

Plots

Confirm that:

- [ ] The axis labels state the marker and fluorochrome used (e.g. CD4-FITC).
- [ ] The axis scales are clearly visible. Include numbers along axes only for bottom left plot of group (a 'group' is an analysis of identical markers).
- [ ] All plots are contour plots with outliers or pseudocolor plots.
- [x] A numerical value for number of cells or percentage (with statistics) is provided.

Methodology

Sample preparation	Cells were fixed with ice-cold 70% ethanol and stored at -20 centigrade. On the day of cell cycle analysis, fixed cells were washed twice with ice-cold PBS and then stained with 50 µg/mL propidium iodide (Invitrogen) in PBS supplemented with 10 µg/mL RNase A (Invitrogen).
Instrument	LSRFortessa flow cytometer (BD Biosciences).
Software	FlowJo ver. 10
Cell population abundance	Since cells were originated from cell lines, cells were considered a homogeneous population of cancer cells.
Gating strategy	Different phases of the cell cycle were gated using FlowJo cell cycle analysis algorithm.

- [x] Tick this box to confirm that a figure exemplifying the gating strategy is provided in the Supplementary Information.
