# Supplementary material for: PRMT5 is an actionable therapeutic target in CDK4/6 inhibitor-resistant ER+/RB-deficient breast cancer
Source: Nat Commun. 2024 Mar 13;15:2287. doi: 10.1038/s41467-024-46495-2 (PMC10937713; doi:10.1038/s41467-024-46495-2)
Supplement: Supplementary file 3 — Description of Additional Supplementary Files [file 41467_2024_46495_MOESM3_ESM.pdf]

Title: Supplementary Data 1. Analysis of the genome-wide CRISPR screen

Description: MAGeCKFlute was used to calculate the β-scores, which represent the degree of sgRNA depletion or enrichment. The relative essentiality for each individual genes was calculated by comparing the difference in the β-scores of T47D_*RB1* knockout (RBKO) and wild-type (WT) cells (β_{RBKO}-β_{WT}).

Title: Supplementary Data 2. Analysis of Co-IP mass spectrometry (PRMT5 pulldowns vs IgG)

Description: Mass spectrometry data for PRMT5 antibody and IgG control pulldowns were acquired using Q-Exactive HF Quadrupole-Orbitrap mass spectrometer (Thermo Fisher). FDR Confidence: High (1% False Discovery Rate). Accession: Protein accession number (from UniProtKB). Description: Description taken from UniProt. Coverage (%): Percentage of the protein sequence that was covered by the peptides identified for the protein. #PSMs: Number of Peptide Spectrum Matches, or the number of spectra assigned to peptides that contributed to the inference of the protein. MW [kDa]: Molecular weight of the protein based on the sequence from UniProt. Abundance: The sum of the peak intensities for each peptide identified for that protein. Statistics were conducted using two-sided Student's t test and then adjusted using Benjamini-Hochberg adjustments (padj) for multiple comparisons.

Supplementary Data 3. Analysis of SDMA PTM scan (siPRMT5 vs siCtrl)

Mass spectrometry data for the SDMA enriched samples were acquired using Orbitrap Fusion Lumos Tribrid mass spectrometer (Thermo Fisher). Abundance: The sum of the peak intensities for each peptide identified for that protein. Statistics were conducted using two-sided Student's t test and then adjusted using Benjamini-Hochberg adjustments (padj) for multiple comparisons.
